# Acute Kidney Injury in Sepsis

**DOI:** 10.3390/ijms25115924

**Published:** 2024-05-29

**Authors:** Telma Pais, Sofia Jorge, José António Lopes

**Affiliations:** Nephrology and Renal Transplantation Department, Unidade Local de Saúde Santa Maria, 1649-028 Lisbon, Portugal; telma.pais@ulssm.min-saude.pt (T.P.);

**Keywords:** acute kidney injury, sepsis-associated acute kidney injury, sepsis-induced acute kidney injury, treatment, sepsis, septic shock

## Abstract

Sepsis-associated kidney injury is common in critically ill patients and significantly increases morbidity and mortality rates. Several complex pathophysiological factors contribute to its presentation and perpetuation, including macrocirculatory and microcirculatory changes, mitochondrial dysfunction, and metabolic reprogramming. Recovery from acute kidney injury (AKI) relies on the evolution towards adaptive mechanisms such as endothelial repair and tubular cell regeneration, while maladaptive repair increases the risk of progression to chronic kidney disease. Fundamental management strategies include early sepsis recognition and prompt treatment, through the administration of adequate antimicrobial agents, fluid resuscitation, and vasoactive agents as needed. In septic patients, organ-specific support is often required, particularly renal replacement therapy (RRT) in the setting of severe AKI, although ongoing debates persist regarding the ideal timing of initiation and dosing of RRT. A comprehensive approach integrating early recognition, targeted interventions, and close monitoring is essential to mitigate the burden of SA-AKI and improve patient outcomes in critical care settings.

## 1. Introduction

Sepsis is defined as a clinical syndrome characterized by life-threatening organ dysfunction due to a dysregulated host response to infection [1]. In septic shock, a type of distributive shock, patients clinically present with sepsis but, despite adequate fluid resuscitation, require vasopressors to maintain a mean arterial pressure (MAP) ≥ 65 mmHg and have a serum lactate level > 2 mmol/L (>18 mg/dL). This subgroup of patients has substantially increased mortality [2].

Both definitions align with the 2016 Third International Consensus (Sepsis-3) Definitions, which aimed to provide more precise terminology for the sepsis spectrum. This was made possible by the growing understanding of the changes in organ function, morphology, biology, and immunology induced by sepsis [2].

Over the past two decades, the criteria for defining acute kidney injury (AKI) have evolved. Currently, the most widely accepted definition of AKI is based on the Kidney Disease: Improving Global Outcomes (KDIGO) guidelines. According to these guidelines, AKI is defined by the presence of any of the following criteria: an increase in serum creatinine (SCr) of at least 0.3 mg/dl within 48 h; an increase in SCr to ≥1.5 times baseline, known or presumed to have occurred within the prior 7 days; or urine output volume < 0.5 mL/kg/h for 6 h [3]. Subsequently, AKI severity is staged based on criteria outlined in Table 1, which consider SCr values and/or urine output volumes [3].

In critically ill patients, sepsis has been associated with 25–75% of reported cases of AKI [4,5,6,7]. While there is currently no universal consensus on the definition for sepsis-associated acute kidney injury (SA-AKI), it has been proposed to entail the presence of both sepsis (as defined by the Sepsis-3 criteria) and AKI (as defined by the KDIGO criteria) [4,8].

This encompasses a wide spectrum of heterogeneous pathophysiological mechanisms, including direct consequences of the infection itself or of the dysregulated host response, and indirect mechanisms that develop as the consequence of sepsis or sepsis therapies (for example, antimicrobial-drug-induced AKI) [4]. In this setting, a sub-phenotype of SA-AKI has been proposed, in which sepsis is the predominant driver of tissue damage, thereby excluding injury as an indirect consequence of either sepsis or its treatment. This sub-phenotype is termed sepsis-induced AKI [4].

Although SA-AKI is common in critically ill patients, estimating its true incidence and prevalence is challenging, primarily due to the lack of a standardized definition for SA-AKI and to the utilization of variable criteria for sepsis and AKI across studies, which compromises data generalization [8]. This challenge was underscored in a systematic review and meta-analysis of 47 observational studies of SA-AKI, which employed three AKI definitions and three diagnostic criteria for sepsis and/or septic shock [6]. Moreover, many co-occurring factors may contribute to AKI development in critically ill patients. The diversity of clinical settings and patient populations, along with the inconsistent reporting of relevant outcomes, may contribute to the knowledge gap.

Overall, AKI is associated with a substantially increased mortality risk during hospitalization (between two- and sixfold) and heightens the likelihood of later progression to chronic kidney disease (CKD) [3,7]. Specifically, SA-AKI carries a greater risk of in-hospital mortality and prolonged hospitalization compared to AKI resulting from other causes. [9].

In this review, we focus on current knowledge regarding the pathophysiology and treatment of SA-AKI.

## 2. Pathophysiology

**Figure 1:**
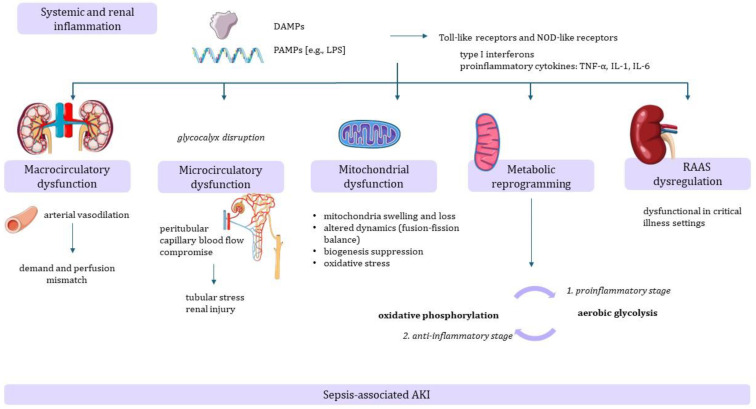
Sepsis-associated acute kidney injury pathophysiology. Abbreviations: AKI, acute kidney injury; DAMPs, damage-associated molecular patterns; IL, interleukin; LPS, lipopolysaccharide; PAMPs, pathogen-associated molecular patterns; NOD- nucleotide oligomerization domain; RAAS, renin–angiotensin–aldosterone system; TNF-α, tumor necrosis factor alpha. (All free elements in the figure originated from Servier Medical Art, http://smart.servier.com (accessed on 14 May 2024). Servier Medical Art by Servier is licensed under a Creative Commons Attribution 4.0 (https://creativecommons.org/licenses/by/4.0).)

SA-AKI arises from complex and heterogenous mechanisms that result in injury. These mechanisms can directly stem from infection and the associated host response, or they may be indirect consequences of sepsis or its treatment. Several pathophysiological mechanisms may interact and lead to AKI in septic patients, including systemic and renal inflammation, microcirculatory dysfunction and macrocirculatory abnormalities, mitochondrial dysfunction, metabolic reprogramming, and dysregulation of the renin–angiotensin–aldosterone system (RAAS) (Figure 1) [4,10].

The initial innate immunity response in sepsis is triggered by exposure to damage-associated molecular patterns, which are endogenous molecules released from damaged cells, and pathogen-associated molecular patterns (such as small molecular motifs, including lipopolysaccharide (LPS) and double-stranded RNA) that serve as ligands for receptors present either on the cell surface, such as Toll-like receptors, or in the cytosol, such as nucleotide oligomerization domain (NOD)-like receptors. The activation of these receptors initiates the transcription and release of type I interferons and proinflammatory cytokines such as tumor necrosis factor alpha (TNF-α), interleukin (IL)-1, and IL-6 [11,12,13,14].

In sepsis, the dysregulated host innate immune response to a pathogen is characterized by an increase in systemic and renal inflammation. There is a hyperexpression of inflammatory markers, increased oxidative stress, and coagulation cascade activation. Proinflammatory mediators activate both the complement system and the innate immunity response [10,12].

Previously, it was believed that the primary driver of the initial injury in SA-AKI was related to the macrocirculatory abnormalities that occur in sepsis, secondary to a demand and perfusion mismatch. Heightened cytokine-induced nitric oxide synthesis leads to generalized arterial vasodilation and decreased systemic vascular resistance, increasing the risk of impaired organ perfusion in the setting of an increased metabolic demand [8,10,15,16]. However, it appears that many more concurrent factors are implicated.

Endothelial dysfunction due to inflammatory and oxidative mediators, along with coagulation disorders and glycocalyx disruption, may contribute to microcirculatory dysfunction, even in the setting of a preserved macrovascular state [8,11,17]. Functional studies using intravital video microscopy in LPS-induced sepsis in mice demonstrated compromise in the peritubular capillary blood flow, contributing to tubular stress and renal injury [18]. The decline in functional capillaries preceded renal dysfunction [18]. Later studies using multiparametric photo-acoustic microscopy (measuring real-time changes in hemoglobin (Hb) concentration, oxygen saturation of Hb, and blood flow in peritubular capillaries in vivo) revealed that sepsis induced a significant reduction in peritubular capillary oxygen saturation of Hb and a decrease in kidney ATP levels, supporting the role of microcirculatory dysfunction in SA-AKI [19]. Therefore, while hemodynamic changes with macrovascular dysfunction may contribute to septic AKI, it can occur independently of renal hypoperfusion [20].

The kidneys have high energetic demands and therefore an important mitochondrial content. In SA-AKI, several mechanisms are involved in the dysregulation of mitochondrial structure (e.g., loss and swelling of mitochondria due to ischemia and opening of mitochondrial permeability transition pores that release pro-apoptotic mediators), dynamics (altered balance between fusion and fission), and biogenesis [21].

Tissue damage in sepsis occurs both as a consequence of direct pathogenic injury and of the dysregulated inflammatory host response to the infection. In SA-AKI, early reprogramming of the metabolic pathways of immune and nonimmune cells can impact the extent of acute and/or chronic organ dysfunction [8,22]. It has been proposed that, in sepsis, tubular epithelial cells undergo an early metabolic switch from oxidative phosphorylation to aerobic glycolysis (via the Akt/mTOR complex 1/hypoxia inducible factor 1α pathway), resulting in an anabolic, proinflammatory stage. Later, there is a switch to oxidative phosphorylation, leading to an anti-inflammatory, catabolic stage.

The initial switch to aerobic glycolysis ensures the reallocation of energy to critical processes, including macromolecule synthesis, while still guaranteeing adequate energy for vital cell functions. It also reduces the generation of reactive oxygen species and, consequently, mitochondrial damage [12]. Additionally, the shift toward aerobic glycolysis is essential for trained immunity, enabling the innate immune system to develop memory and modify the response to future injury [22].

On the other hand, persistence of a proinflammatory response leads to a cyclic trend of tissue injury and organ dysfunction. The ability to revert metabolism back to oxidative phosphorylation is of utmost importance in obtaining adequate inflammatory de-escalation, helping to prevent chronic inflammation that might lead to kidney fibrosis and CKD [12,22].

The renin–angiotensin–aldosterone system (RAAS) plays a crucial role in blood pressure management, fluid and electrolyte homeostasis, and glomerular filtration rate regulation. This system is believed to be dysfunctional in critical illness settings, such as sepsis. In a multicenter study of 280 critically ill patients with (at least stage 2) AKI or without AKI, elevated serum renin levels were associated with twice as many major adverse kidney events at hospital discharge [23].

Additional indirect factors may exacerbate kidney injury, such as exposure to nephrotoxic drugs and abdominal compartment syndrome. A multicenter prospective descriptive cohort study conducted in ten intensive care unit settings reported that 62% of patients received at least one nephrotoxic drug, with one-third receiving two or more [24].

The complex interplay of various pathophysiological mechanisms in vivo culminates in the multifaceted presentation of SA-AKI.

Later, the trajectory towards recovery from AKI is influenced by several factors, including genetic susceptibility, pre-existing comorbidities, underlying CKD, and the severity of AKI [4,8].

The ability to restore physiological renal processes is dependent on transitioning towards adaptive mechanisms of repair, which involve endothelial restoration, as well as the proliferation and re-differentiation of tubular epithelial cells [4]. If, on the contrary, endothelial-to-mesenchymal transition occurs, along with loss of peritubular capillary density, interstitial fibrosis, and tubular atrophy, a pattern of maladaptive repair emerges. This latter scenario increases the risk of CKD development, particularly in cases of recurrent injury.

## 3. Treatment

Prevention of SA-AKI is challenging, as most patients who develop SA-AKI will already have it at presentation. Several factors that increase the risk of SA-AKI, such as pre-existing chronic diseases (e.g., hypertension and diabetes), source, and severity of infection, cannot be changed [6]. Therefore, the main goals reside in early recognition of sepsis, given it is a medical emergency, and treatment initiation [1,8].

General care measures and AKI-specific therapies should be implemented as appropriate in the clinical setting. The most common interventions include timely administration of appropriate antimicrobial drugs achieving control or elimination of the source of infection, along with fluid resuscitation, vasoactive drugs, and other organ-support measures (e.g., mechanical ventilation or renal replacement therapy [RRT]) as required.

### 3.1. Antimicrobial Administration

If sepsis is suspected, cultures should be collected, and antimicrobials administered immediately, ideally within one hour of presentation [1]. Selection of antimicrobial drugs should be guided by several factors, including patient history and comorbidities, symptoms suggestive of the likely site of infection, underlying immune defects, presence of invasive devices, and local prevalence and resistance patterns. Source control measures include all actions intended to control or remove the foci of infection and to restore its optimal function, and it remains a cornerstone of sepsis treatment [25,26].

Nephrotoxic agents (e.g., aminoglycosides) should be avoided whenever possible. Careful consideration of the specific clinical context and a risk–benefit assessment should be undertaken on an individual basis.

Antimicrobial de-escalation should be routinely considered based on biological sample culture results and clinical evolution. If feasible, drugs should be switched to agents with lower nephrotoxic risk [1]. Monitoring of plasma drugs levels is advised, taking into account its pharmacokinetic/pharmacodynamic properties to avoid unnecessarily high, toxic levels [1].

### 3.2. Fluid Resuscitation

Immediate administration of intravenous fluids is a critical step in sepsis management to restore intravascular volume and maintain adequate perfusion and tissue oxygen delivery [1,11]. Current sepsis recommendations advocate for starting resuscitation with a bolus of fluid (at least 30 mL/kg within the first three hours) [1]. Continuous monitoring of the patient’s response to fluid resuscitation, ideally using dynamic measures, is necessary to prevent over-resuscitation and under-resuscitation, both of which have been linked to adverse outcomes [1].

Several studies have shown that positive fluid balance during and after AKI is an important factor associated with increased mortality [27,28]. This is believed to occur due to endothelial dysfunction, increased cardiac overload compromising cardiac output and renal perfusion, and increased intra-abdominal from tissue edema that may impair renal venous drainage [29].

In cases of insufficient response to initial fluid resuscitation, additional fluids or vasoactive drugs are warranted to prevent further clinical deterioration.

Sepsis-3 guidelines recommend using crystalloids as first-line fluid for resuscitation, with weak strength of evidence favoring balanced crystalloids over 0.9% sodium chloride solution [1]. However, although balanced crystalloids are often used in this setting, no clinical benefit of balanced solutions over the use of 0.9% saline was reported in the BaSICS and PLUS trials and meta-analysis [30,31]. Albumin use is advised only in patients who receive large volumes of crystalloids, and the use of starches and gelatin is discouraged [1].

A protocol-based management approach, consisting of a 6 h resuscitation protocol for the administration of intravenous fluids, vasopressors, inotropes, and red-cell transfusion to achieve prespecified targets, has been proposed as a systematic approach to septic shock patients. Nevertheless, three major randomized clinical trials comparing protocol-based management versus standard care did not demonstrate improved outcomes for septic shock patients regarding mortality or need for RRT [32,33,34,35].

### 3.3. Vasoactive Drugs

Several vasoactive drugs are available when mean arterial pressure (MAP) remains inadequate despite resuscitation measures, such as in the setting of septic shock. Several large-scale multicenter trials have attempted to determine which subset of patients may benefit from the use of drugs such as norepinephrine (noradrenaline), epinephrine, vasopressin, and dopamine, along with angiotensin II and levosimendan [36,37,38,39]. International guidelines recommend using norepinephrine as the first-line agent; if ineffective, adding vasopressin instead of escalating the dose of norepinephrine is advised [1].

This recommendation stems from several clinical trials suggesting better outcomes and/or fewer adverse events with norepinephrine compared with other vasoactive drugs [38,40,41].

Treatment should be individualized, considering patient characteristics and hemodynamic parameters, and, when possible, invasive monitoring of arterial blood pressure is preferred [1].

Current Surviving Sepsis Campaign guidelines recommend an initial target MAP value of at least 65 mmHg in patients with septic shock [1]. However, in patients with pre-existing hypertension, the optimum MAP goal has been a subject of debate, as a rightward shift of the curve for organ pressure-flow autoregulation is expected, and a higher MAP could theoretically better ensure tissue perfusion [42,43].

Other guidelines underscore this, recommending individualizing the target blood pressure and, while they maintain the initial recommendation for MAP values ≥ 65 mmHg, suggest a higher MAP target in septic patients with history of hypertension and in patients that show clinical improvement with higher blood pressure [44].

A multicenter randomized clinical trial (RCT) evaluating 776 patients with septic shock under volume resuscitation with a MAP target of either 80 to 85 mm Hg (high-target group) or 65 to 70 mm Hg (low-target group) found no significant differences in mortality at either 28 or 90 days [45]. Another recent meta-analysis of three RCT trials that recruited 3357 patients also reported no significant difference in all-cause mortality between the lower (60–70 mmHg) and higher (>70 mmHg) MAP target groups. Nevertheless, in the higher MAP target group of patients with chronic hypertension, there was less need for RRT, suggesting potential renal benefit in these patients [46].

### 3.4. Renal Replacement Therapy

Organ-specific support is often necessary in septic patients. For patients with severe AKI, RRT may be required, and the emergent indications for initiating RRT do not differ between SA-AKI and other types of acute kidney injury [3,4]. Nevertheless, the timing and dose of RRT are ongoing sources of debate.

Several studies have evaluated the timing of RRT initiation and, initially, some suggested a survival advantage with early initiation of RRT in patients with severe AKI [47,48]. In the early versus late initiation of RRT in critically ill patients with AKI (ELAIN) trial, a single-center randomized clinical trial, 231 critically ill patients with AKI stage 2 and plasma-neutrophil-gelatinase-associated lipocalin level higher than 150 ng/mL were randomized to early or delayed initiation of RRT. The early RRT start strategy reduced 90 day mortality, as well as length of hospital stay [49]. Contrastingly, the Artificial Kidney Initiation for Kidney Injury (AKIKI) trial, which randomized 620 patients with severe AKI requiring mechanical ventilation and/or catecholamine infusion to either early or delayed strategy of RRT, found no significant difference in 60 day mortality [50].

More recently, in the Initiation of Dialysis Early Versus Delayed in the Intensive Care Unit (IDEAL-ICU) trial involving 477 patients with septic shock and severe AKI, no significant difference in 90 day mortality was found between early versus delayed RRT strategies [51]. Similarly, in a multinational RCT involving 2927 critically ill patients with severe acute kidney injury (STARRT-AKI), over half of whom with sepsis, no mortality benefit was found at 90 days with an accelerated RRT strategy versus standard strategy [52].

Initiating RRT too early might unnecessarily subject patients who would recover renal function with conservative treatment alone to potential RRT side-effects [50]. In IDEAL-ICU, 29% of the patients in the delayed-strategy group did not require RRT because they experienced spontaneous recovery of renal function [51]. More adverse effects were reported in the groups with early RRT initiation, such as hypotension, hypophosphatemia, and catheter-related nosocomial infections [50,52].

A Cochrane meta-analysis, predominantly based on low-quality evidence, identified less consistent findings. The absence of high-quality evidence of efficacy and the risk of increased adverse events led to the conclusion against the routine use of early RRT in critically ill patients with AKI [53].

Therefore, regarding the timing of RRT initiation, evidence seems to point to no benefit (and potential harm) with earlier initiation. A watchful waiting strategy appears to be safe, and recent meta-analyses have confirmed the efficacy of this approach [54,55].

Nonetheless, in patients with severe AKI, careful monitoring is of utmost importance to guarantee that if a conventional indication for RRT develops, it is identified quickly to avoid adverse outcomes such as preventable complications and death. Strategies such as dynamic renal function assessment, focused on dynamic assessment of renal demand–capacity matching and renal biomarkers have been proposed to aid in monitoring and deciding whether RRT should be initiated [54].

The RRT dose in the setting of SA-AKI has been examined, with current evidence supporting a recommended delivery dose of 20–25 mL/kg/h in continuous RRT [56,57]. Several studies have failed to demonstrate benefits from increased dosing of RRT [58,59,60].

Regarding the choice between intermittent versus continuous RRT, no difference has been demonstrated [61]. Therefore, both modalities may be used in the setting of SA-AKI, as long as there is an adequate delivered dose of 20–25 mL/kg/h (continuous RRT) or a Kt/V of 3.9 per week (intermittent dialysis) [62,63]. Technique availability and personnel experience may be important factors to consider.

### 3.5. Extracorporeal Therapies

The use of extracorporeal therapies to remove circulating endotoxin has been studied in the setting of septic shock. Theoretically, removal of substances such as circulating endotoxin and/or cytokines could impact favorably reduce the perpetuation of septic mechanisms, but currently there is not enough evidence to recommend its use in the setting of septic shock or SA-AKI [64,65,66].

### 3.6. Mechanical Ventilation

Mechanical ventilation may be necessary in some patients with sepsis and septic shock for adequate oxygenation, ventilation, and/or airway patency purposes. In this setting, there is an increased risk for AKI, up to threefold [67,68]. Several factors are involved, through mechanical, neurohormonal, and inflammatory mechanisms.

Mechanical factors arise from the use of positive pressure ventilation (PPV), which increases intrathoracic pressure, causing a decrease in cardiac output by reducing venous return. This mechanism, initially demonstrated in canine models, accounts for a posterior decrease in renal perfusion [69]. Additional mechanisms are involved: during mechanical ventilation, deleterious neurohormonal mediators act via sympathetic stimulation and the renin–angiotensin system, leading to changes in the intrarenal blood flow from cortex to medulla. This results in a reduction in the glomerular filtration rate and increased sodium reabsorption, which demands increased oxygen use, worsening the oxygen supply–demand balance [62,68].

A third contributing factor occurs through the systemic release of inflammatory mediators from the lung in the setting of ventilator-induced lung injury (VILI) [70]. Damage to the pulmonary epithelial and endothelial cells activates the innate immune cascade, resulting in the release of pro-inflammatory cytokines, such as IL-6 and TNF-α. This exacerbates the inflammatory cascade already present in the genesis and perpetuation of SA-AKI.

In particular, the use of high tidal volumes in PPV seems to increase the risk of barotrauma and/or volutrauma and therefore the risk of VILI. Experiments using serum from rabbits subjected to injurious, high tidal volume mechanical ventilation were able to induce in vitro epithelial cell apoptosis in the kidney [71].

In many critically ill septic patients, mechanical ventilation is unavoidable. Measures such as avoiding the use of high tidal volumes in PPV, if feasible according to the patient’s clinical status, attempt to reduce the risk of AKI associated with mechanical ventilation.

## 4. Conclusions

SA-AKI can best be defined by concurrent presence of consensus sepsis and AKI criteria and has a complex pathophysiological background that is still not fully understood. Factors such as systemic and renal inflammation, mitochondrial dysregulation, microcirculatory dysfunction, microcirculatory changes, metabolic reprogramming, and RAAS dysfunction have been reported. Prompt diagnosis and treatment guided by best practice guidelines are crucial, as there are usually no timely and effective prevention measures available. Early administration of antimicrobial drugs, source control, and fluid resuscitation, along with vasoactive drugs and organ-support measures as needed are advised.

Further research into underlying mechanisms may help enlighten the impact that inter-individual variability may have on response to treatment, with the goal of effectively treating SA-AKI and improving its prognosis.

## Figures and Tables

**Table 1:** Staging of AKI according to KDIGO guidelines.

	Serum Creatinine Criteria	Urine Output
Stage 1	SCr 1.5 to 1.9 times the baseline value OR SCr ≥0.3 mg/dL (≥26.5 µmol/L)	<0.5 mL/kg/h for 6–12 h
Stage 2	SCr 2.0 to 2.9 times baseline	<0.5 mL/kg/h for ≥12 h
Stage 3	SCr rises to 3.0 times baseline OR Increase in SCr to ≥4.0 mg/dL (≥354 µmol/L) OR Need for initiation of renal replacement therapy	<0.3 mL/kg/h for ≥24 h OR Anuria for ≥12 h

## Data Availability

No new data were created or analyzed in this study. Data sharing is not applicable to this article.
